# Cause of death in people living with HIV who initiated antiretroviral therapy after enrolling to the Thai National AIDS Program from 2008 to 2021

**DOI:** 10.1016/j.lansea.2025.100576

**Published:** 2025-04-23

**Authors:** Cheewanan Lertpiriyasuwat, Stephen J. Kerr, Sairat Noknoy, Patiphak Namahoot, Niramon Punsuwan, Tanakorn Apornpong, Jiratchaya Sophonphan, Napon Hiranburana, Ploenchan Chetchotisakd, Opass Putcharoen, Kiat Ruxrungtham, Anchalee Avihingsanon

**Affiliations:** a Division of AIDS and STIs, Ministry of Public Health, Nonthaburi 11000 Thailand; b HIV-NAT, Thai Red Cross AIDS and Infectious Diseases Research Centre (formerly Known as the Thai Red Cross AIDS Research Centre (TRCARC)), Bangkok 10330 Thailand; c Faculty of Medicine, Research Affairs, Chulalongkorn University, Bangkok 10330 Thailand; d The Kirby Institute, The University of New South Wales, Sydney 2033 Australia; e National Health Security Office, Nonthaburi 11000 Thailand; f Faculty of Medicine, Center of Excellence in Tuberculosis, Chulalongkorn University, Bangkok 10330 Thailand; g Department of Medicine, Faculty of Medicine, Srinagarind Hospital, Khon Kaen University, Khon Kaen 40002 Thailand; h Division of Infectious Diseases, Department of Medicine, Faculty of Medicine, Chulalongkorn University, Bangkok 10330 Thailand; i Thai Red Cross Emerging Infectious Diseases Clinical Centre, King Chulalongkorn Memorial Hospital, Bangkok 10330 Thailand; j School of Global Health, Faculty of Medicine, Chulalongkorn University, Bangkok 10330 Thailand; k Faculty of Medicine, Centre of Excellence in Vaccine Research and Development (Chula VRC), Chulalongkorn University, Bangkok 10330 Thailand

**Keywords:** Cause of death, People living with HIV, Thailand National AIDS Program

## Abstract

**Background:**

Widespread access to antiretroviral therapy (ART) has led to near-normal life expectancies for people living with HIV (PLHIV), shifting the leading cause of death (COD) from AIDS-related to non-AIDS-related mortality. We assessed trends in COD among PLHIV who initiated ART in Thai National AIDS Program (NAP).

**Methods:**

We analysed NAP data from PLHIV aged ≥15 at ART initiation, who started ART under Thailand’s universal health coverage from 2008 to 2021. Individual data was linked with the National Death Registration system, and a rule-based algorithm applied text mining to classify COD as AIDS-related, non-AIDS-related and uncertain. Competing risk models examined associations between demographic and clinical characteristics and COD. Standardized mortality ratios (SMR) were calculated using mortality rate from the general Thai population.

**Findings:**

Among 398,182 PLHIV (37.1% females) enrolled, the median (IQR) age was 35 (28–43) years, 43.6% commenced ART with CD4 counts <200 cells/mm3. Over 2,631,435 person years of follow-up, 73,768 (18.5%) deaths occurred: 56% AIDS-related, 40% non-AIDS-related and 4% uncertain. The cumulative incidence of AIDS-related mortality at 14 years was 14.74%, non-AIDS-related 12.04% and all-cause mortality 27.93%. AIDS-related deaths declined from 60% to 50% over the study period. Low CD4 counts, permanently loss to care and treatment at non-capital city were significantly associated with higher AIDS-related mortality. The SMR was higher in females [9.08 (95% CI 8.97–9.20] compared to males [5.83 (95% CI 5.78–5.88).

**Interpretation:**

AIDS-related mortality decreased over time, but continued efforts are needed to improve earlier diagnosis, and equitable outcomes for women and those residing outside major cities.

**Funding:**

Supported by National Institute of Health (IeDEA:U01AI069907).

**Research in context**

**Evidence before this study**

We searched PubMed with the terms “cause of death” or “sex-differences” and “HIV” or antiretroviral therapy”. Life expectancy for PLHIV who access antiretroviral therapy with high CD4 counts is comparable to the general population and causes of death in PLHIV have shifted from those associated with AIDS, to non-communicable diseases associated with ageing. Standardized mortality ratios remain higher for PLHIV than the general population. There is a strong relationship between increased mortality and lower CD4 counts at ART start, failure to suppress viral load and in high burden TB countries, TB remains a leading cause of death.

**Added value of this study**

Our population-based study demonstrates that despite free health care and interventions targeted to key populations, there are inequalities in Thailand whereby people living outside the capital city present to the health system with more advanced HIV disease and experience higher mortality from both AIDS- and non-AIDS related causes. Notably, women living with HIV are more likely to die compared to the general population than men, despite presenting to care with higher CD4 counts than males, and the observed sex-differences are more pronounced for younger women. Adolescents are at a high risk of AIDS related deaths.

**Implications of all the available evidence**

The findings highlight the urgent need to explore the underlying causes of regional and sex-related disparities in health outcomes. Targeted interventions must be developed to specifically address the root causes of these inequities, ensuring equitable HIV treatment outcomes for women and men, and all populations across Thailand, independent of where they reside. Given the high rate of late presenters in this cohort, it is crucial to enhance advanced HIV disease management to prevent avoidable deaths. Additionally, strategies for early diagnosis and prompt treatment of HIV should be prioritized to improve outcomes for this population.

## Introduction

Access to effective antiretroviral therapy (ART) and laboratory testing through universal health coverage (UHC) has significantly increased life expectancy and quality of life for people living with HIV (PLHIV) in Thailand.^1^^,^^2^ Thailand's national health programs have greatly reduced the barriers to HIV care by ensuring that HIV treatment and care are widely accessible, including marginalized and rural populations. Recent studies in high income countries have documented a shift in the cause of death (COD) for PLHIV, moving from primarily AIDS-related death to non-communicable diseases (NCDs), at rates higher than the general population.^3^, ^4^, ^5^ These NCDs include cardiovascular diseases, liver diseases, and cancers, reflecting the epidemiological transition driven by the ageing HIV-positive population, long-term ART exposure and chronic, low level immune activation, even in individuals who achieve viral suppression. Similar trends have been observed in other high income Asian countries including Japan,^6^ where the ageing HIV cohort is also experiencing an increase in NCDs as the leading causes of death. However, there are limited data available from low-middle income countries (LMICs) in Asia, including Thailand, where healthcare infrastructure, socioeconomic factors, and the epidemiological profile of HIV may differ significantly from those in higher-income settings.

In Thailand, ART initiation and care are offered under the country’s UHC scheme since 2008, providing an opportunity to study mortality patterns and identify factors influencing outcomes. One of the strongest predictors of subsequent mortality in PLHIV is the CD4 count at the time of ART initiation.^7^ Early initiation of ART at higher CD4 counts is associated with better survival outcomes, as it helps maintain a higher level of immune function and reduces the risk of opportunistic infections and AIDS-related complications. However, despite the availability of ART, late diagnosis and initiation of treatment remain a major public health challenge, with substantial AIDS-related mortality still occurring among those who present with advanced HIV disease and low CD4 counts.^8^^,^^9^ In this context, understanding the specific causes of death among PLHIV in Thailand is essential for optimizing clinical care and public health strategies. This study aimed to describe the COD in PLHIV who initiated antiretroviral therapy after enrolling in Thailand’s National AIDS Program (NAP) from 2008 to 2021. Analysing these patterns will identify areas for improvement in the management of HIV and associated conditions, and potentially inform policy decisions, and target interventions to specific groups to reduce mortality in this population.

## Methods

We conducted a retrospective analysis of NAP database of PLHIV who initiated ART after registering to the NAP between 2008 and 2021. The cohort included individuals aged 15 years or older at ART initiation. During this period, the preferred first-line regimen was efavirenz with two nucleoside-reverse transcriptase inhibitors. CD4 thresholds for initiating ART in National Treatment Guidelines were <200 cells/mm^3^ until 2010, <350 cells/mm^3^ from 2011 to 2013 and at any CD4 count from 2014 onwards. The NAP has collected demographic, HIV-related data and laboratory data for all PLHIV under UHC in Thailand since 2008. This program ensures that all individuals, regardless of their socioeconomic status or geographic location, have access to HIV care and treatment.

The NAP database is managed by the National Health Security Office (NHSO), and used to reimburse facilities for treatment, laboratory testing and care provided to health care clients. The Thai law mandates that deaths be reported to the Ministry of the Interior within 24 h, and since 2008, deaths have been electronically linked to the NHSO database using a citizen’s National Identity Card number. Originally, linkages were updated twice monthly, but in recent years, the deaths are updated in real time. The NAP links individual data with the National Death Registration system with non-coded, free-text data documenting causes of death. We developed a rule-based algorithm which applied text mining to classify COD from free-text on death certificates. Free text was categorised into 3 major groups: 1) AIDS-related causes (AIDS defining infection and cancers/other and AIDS unspecified COD); 2) non-AIDS related causes including non-communicable diseases and non-natural causes, and 3) uncertain COD, following the Thai HIV case surveillance definitions and US CDC revised classification system.^10^^,^^11^ Accuracy evaluation involved expert reviews of both free-text and algorithm-derived causes of a random sample of 544 deaths.

The study was approved by the Institutional Review Board (IRB) of the Institute for Development of Human Research Protection, Ministry of Public Health (MOPH), Thailand (No. 589/2554). A waiver of consent was granted, and all data were deidentified by the NHSO before analysis.

### Role of the funding source

The funding bodies had no role in any part of the study.

### Statistical analysis

Statistical analysis was performed using Stata 18.5 (Statcorp LLC, College Station, TX, USA). Participant demographics were described by year of enrolment to the NAP program. PLHIV who remained alive had their follow-up censored on 30 January 2023. The crude cumulative incidence (CI) of each broadly classified COD, with other COD as competing risks, was calculated using non-parametric methods (stcompet command in Stata) and plotted for different covariates. Due to the size of the dataset, we reformatted the data with time dependent weights using stcrprep^12^ which enables the Fine and Gray proportional sub-hazards model to be fit using standard Stata survival analysis commands in a computationally efficient way. Adjusted sub-distribution hazard ratios (sHR) for AIDS-related and non-AIDS related COD were calculated. Factors adjusted for in competing risk models were age, sex, CD4 count at ART start, CDC category C disease at ART start, geographical region of the country, whether participants were permanently lost from care and period of ART initiation corresponding to the Thai National Treatment Guideline according to the CD4 criteria (2008–2010, 2011–2013 and 2014 onwards). Age was categorised as 15–19, 20–29, 30–39, 40–49 and ≥ 50 years, CD4 count was categorised as <50, 50–99, 100–199, 200–349, 350–499, ≥500 and unknown, permanently lost to care was defined as a gap in clinic attendance for ≥365 days and never returning to care. CD4 count at ART start was defined as the closest CD4 count within a window ranging from 1 year before ART start, to 3 months after starting ART. Hazard ratios for all-cause mortality were also calculated using Cox regression. The proportional hazards assumption was evaluated by overlaying the observed versus model predicted survival curves on the same graph. Since the assumption of proportional sub-hazards and proportional hazards was violated if cohort participants were lost, our models were generated after stratifying the analysis on this variable. Lastly, we calculated the standardized mortality ratios (SMR) overall, by sex, and CD4 category at ART initiation, using data from the Thai general population from the Thailand official registration systems’ website as a reference.^13^

## Results

### Cohort description

**Fig. 1:**
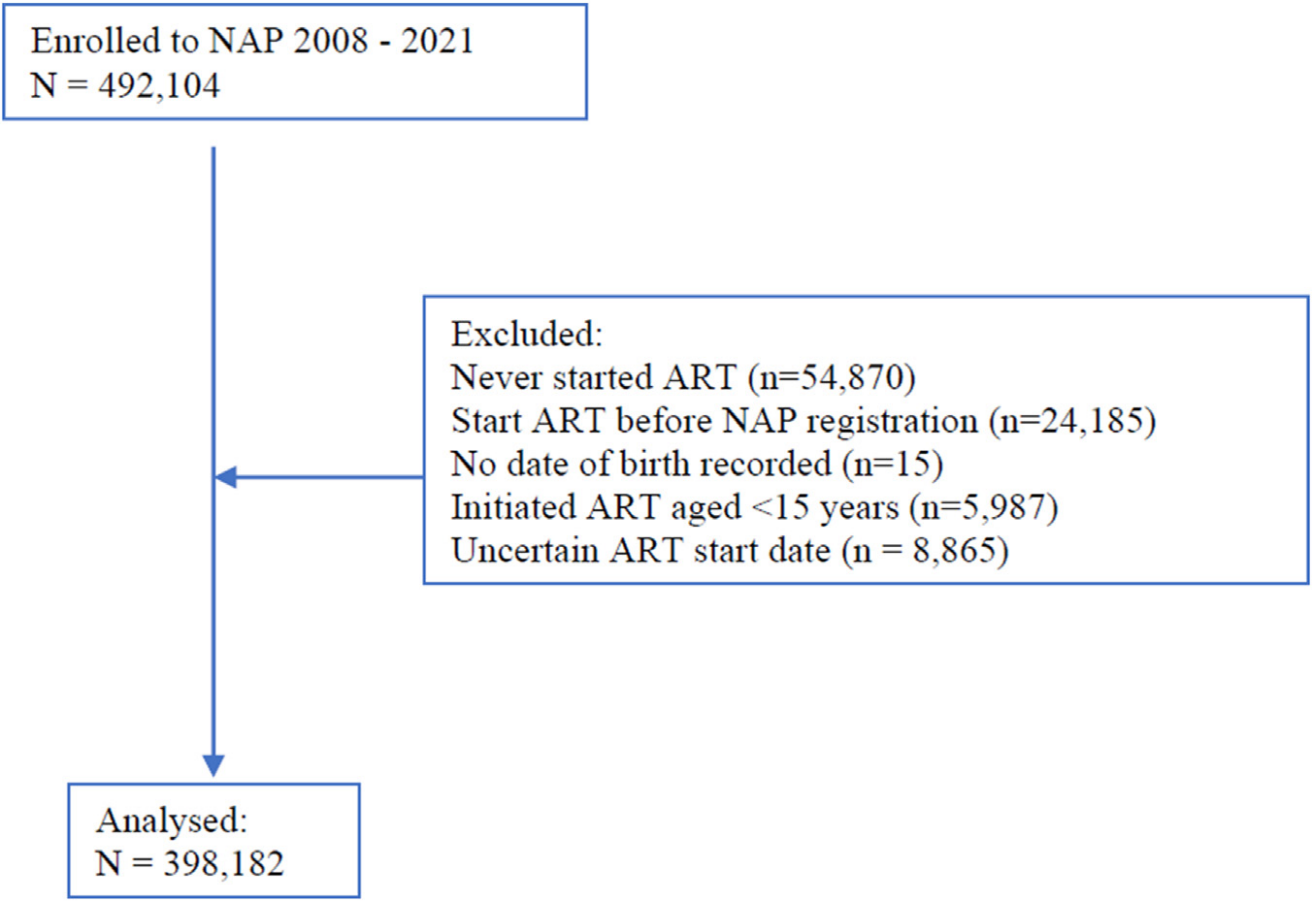
Study disposition.

A total of 492,104 PLHIV were registered into the NAP from 2008 to 2021. After excluding 54,870 who did not start ART before 30 January 2023, 24,185 initiated ART before registration into the NAP, the birth date was not recorded for 15 people, 5987 were under 15 years old at ART initiation, and 8865 had uncertain ART start date, so our final analysis dataset was comprised of 398,182 PLHIV (Fig. 1). Characteristics of the participants by year of program enrolment are shown in Table 1. Over time, the median (IQR) duration from program enrolment to ART initiation decreased from 81 (20–630) days in 2008, to 14 (2–35) days in 2021. During follow-up, 147,833 (37.1%) PLHIV who started ART were female; the percentage of females who were registered into the NAP program was approximately 50% in 2008 and gradually decreased to 28% in 2021. Median (IQR) age at ART start over total follow-up was 35 (28–43) years and ranged from 33 to 38 over the study period. Median (IQR) age at ART start was 37 (29–44) years in women and 35 (27–42) years for men. The earlier years had higher numbers of participants with no CD4 count within the window defined for baseline (19.6% in 2008). This gradually decreased but rose again reflecting the changes within the guidelines which recommended ART initiation at any CD4 count. Over the total enrolment period, 76,201 (19.1%) commenced ART with CD4 counts <50 cells/mm^3^, 173,797 (43.6%) commenced ART with CD4 counts <200 cells/mm^3^ and 243,029 (61.0%) started ART with CD4 counts <350 cells/mm^3^. Median CD4 cell count at ART start was 213 (69–409) cells/mm^3^ in women and 180 (53–369) cells/mm^3^ in men.

**Table 1:** Participant characteristics by year of NAP registration.

N	Year of program registration														
	2008	2009	2010	2011	2012	2013	2014	2015	2016	2017	2018	2019	2020	2021	Total
	33,556 (8.4%)	31,964 (8.0%)	29,922 (7.5%)	28,448 (7.1%)	32,422 (8.1%)	36,247 (9.1%)	29,529 (7.4%)	29,003 (7.3%)	27,073 (6.8%)	26,663 (6.7%)	26,507 (6.7%)	24,354 (6.1%)	23,378 (5.9%)	19,116 (4.8%)	398,182 (100.0%)
**Median (IQR) age at ART start [years]**	36 (31–42)	36 (31–43)	36 (30–43)	36 (30–43)	37 (30–44)	38 (30–45)	35 (28–43)	35 (27–43)	34 (26–43)	33 (25–43)	33 (26–43)	33 (25–43)	34 (26–44)	33 (25–43)	35 (28–43)
**Age category (years)**															
15–19	456 (1.4%)	543 (1.7%)	668 (2.2%)	824 (2.9%)	841 (2.6%)	970 (2.7%)	1195 (4.0%)	1339 (4.6%)	1527 (5.6%)	1567 (5.9%)	1497 (5.6%)	1401 (5.8%)	1250 (5.3%)	1039 (5.4%)	15,117 (3.8%)
20–29	6037 (18.0%)	5945 (18.6%)	5802 (19.4%)	5793 (20.4%)	6474 (20.0%)	7234 (20.0%)	7854 (26.6%)	8366 (28.8%)	8093 (29.9%)	8699 (32.6%)	8756 (33.0%)	8236 (33.8%)	7566 (32.4%)	6398 (33.5%)	101,253 (25.4%)
30–39	15,479 (46.1%)	13,968 (43.7%)	12,483 (41.7%)	11,220 (39.4%)	12,304 (37.9%)	12,509 (34.5%)	9656 (32.7%)	9165 (31.6%)	8263 (30.5%)	7596 (28.5%)	7536 (28.4%)	6735 (27.7%)	6404 (27.4%)	5201 (27.2%)	138,519 (34.8%)
40–49	8727 (26.0%)	8535 (26.7%)	8039 (26.9%)	7660 (26.9%)	9175 (28.3%)	10,643 (29.4%)	7391 (25.0%)	6749 (23.3%)	6104 (22.5%)	5742 (21.5%)	5601 (21.1%)	4888 (20.1%)	4906 (21.0%)	3863 (20.2%)	98,023 (24.6%)
≥50	2857 (8.5%)	2973 (9.3%)	2930 (9.8%)	2951 (10.4%)	3628 (11.2%)	4891 (13.5%)	3433 (11.6%)	3384 (11.7%)	3086 (11.4%)	3059 (11.5%)	3117 (11.8%)	3094 (12.7%)	3252 (13.9%)	2615 (13.7%)	45,270 (11.4%)
**Sex**															
Female	16,413 (48.9%)	14,670 (45.9%)	13,283 (44.4%)	12,434 (43.7%)	13,780 (42.5%)	14,044 (38.7%)	10,455 (35.4%)	9561 (33.0%)	8496 (31.4%)	7966 (29.9%)	7712 (29.1%)	6818 (28.0%)	6848 (29.3%)	5353 (28.0%)	147,833 (37.1%)
Male	17,143 (51.1%)	17,294 (54.1%)	16,639 (55.6%)	16,014 (56.3%)	18,642 (57.5%)	22,203 (61.3%)	19,074 (64.6%)	19,442 (67.0%)	18,577 (68.6%)	18,697 (70.1%)	18,795 (70.9%)	17,536 (72.0%)	16,530 (70.7%)	13,763 (72.0%)	250,349 (62.9%)
**CD4 count at ART start [cells/mm3]**															
<50	7083 (21.1%)	6928 (21.7%)	6338 (21.2%)	5787 (20.3%)	5992 (18.5%)	6397 (17.6%)	5907 (20.0%)	5465 (18.8%)	4885 (18.0%)	4706 (17.6%)	4752 (17.9%)	4234 (17.4%)	4090 (17.5%)	3637 (19.0%)	76,201 (19.1%)
50–99	3503 (10.4%)	3551 (11.1%)	3331 (11.1%)	3073 (10.8%)	3306 (10.2%)	3287 (9.1%)	3050 (10.3%)	2960 (10.2%)	2753 (10.2%)	2506 (9.4%)	2332 (8.8%)	2134 (8.8%)	2104 (9.0%)	1775 (9.3%)	39,665 (10.0%)
100/199	5958 (17.8%)	5316 (16.6%)	4693 (15.7%)	4237 (14.9%)	4751 (14.7%)	4662 (12.9%)	4283 (14.5%)	4127 (14.2%)	3749 (13.8%)	3579 (13.4%)	3615 (13.6%)	3358 (13.8%)	3134 (13.4%)	2469 (12.9%)	57,931 (14.5%)
200–349	5280 (15.7%)	5019 (15.7%)	4925 (16.5%)	4674 (16.4%)	5871 (18.1%)	6435 (17.8%)	5409 (18.3%)	5147 (17.7%)	4766 (17.6%)	4772 (17.9%)	4928 (18.6%)	4563 (18.7%)	4147 (17.7%)	3296 (17.2%)	69,232 (17.4%)
350–499	2590 (7.7%)	2651 (8.3%)	2700 (9.0%)	2673 (9.4%)	3843 (11.9%)	4597 (12.7%)	3550 (12.0%)	3825 (13.2%)	3724 (13.8%)	3711 (13.9%)	3651 (13.8%)	3390 (13.9%)	3152 (13.5%)	2253 (11.8%)	46,310 (11.6%)
≥500	2574 (7.7%)	2637 (8.2%)	2594 (8.7%)	2631 (9.2%)	4145 (12.8%)	6266 (17.3%)	3925 (13.3%)	4252 (14.7%)	4073 (15.0%)	4261 (16.0%)	4007 (15.1%)	3674 (15.1%)	3369 (14.4%)	2325 (12.2%)	50,733 (12.7%)
Unknown	6568 (19.6%)	5862 (18.3%)	5341 (17.8%)	5373 (18.9%)	4514 (13.9%)	4603 (12.7%)	3405 (11.5%)	3227 (11.1%)	3123 (11.5%)	3128 (11.7%)	3222 (12.2%)	3001 (12.3%)	3382 (14.5%)	3361 (17.6%)	58,110 (14.6%)
**CDC Category at ART start**															
CDC A/B	19,714 (58.7%)	19,604 (61.3%)	20,010 (66.9%)	19,619 (69.0%)	21,940 (67.7%)	23,487 (64.8%)	20,767 (70.3%)	21,060 (72.6%)	20,573 (76.0%)	20,613 (77.3%)	21,039 (79.4%)	19,767 (81.2%)	18,053 (77.2%)	15,377 (80.4%)	281,623 (70.7%)
CDC category C	9410 (28.0%)	9606 (30.1%)	8511 (28.4%)	8535 (30.0%)	10,459 (32.3%)	12,737 (35.1%)	8748 (29.6%)	7933 (27.4%)	6491 (24.0%)	6046 (22.7%)	5466 (20.6%)	4585 (18.8%)	4215 (18.0%)	3350 (17.5%)	106,092 (26.6%)
Unknown	4432 (13.2%)	2754 (8.6%)	1401 (4.7%)	294 (1.0%)	23 (0.1%)	23 (0.1%)	14 (0.0%)	10 (0.0%)	9 (0.0%)	4 (0.0%)	2 (0.0%)	2 (0.0%)	1110 (4.7%)	389 (2.0%)	10,467 (2.6%)
**Region**															
Bangkok	3538 (10.5%)	3764 (11.8%)	3726 (12.5%)	3650 (12.8%)	6353 (19.6%)	9173 (25.3%)	5452 (18.5%)	5547 (19.1%)	4975 (18.4%)	5142 (19.3%)	5119 (19.3%)	4849 (19.9%)	4405 (18.8%)	3236 (16.9%)	68,929 (17.3%)
Central	4904 (14.6%)	5031 (15.7%)	4568 (15.3%)	4552 (16.0%)	5014 (15.5%)	7111 (19.6%)	5319 (18.0%)	5056 (17.4%)	4799 (17.7%)	4672 (17.5%)	4581 (17.3%)	4036 (16.6%)	3637 (15.6%)	2966 (15.5%)	66,246 (16.6%)
Eastern	3535 (10.5%)	3754 (11.7%)	3460 (11.6%)	3378 (11.9%)	3799 (11.7%)	3424 (9.4%)	3336 (11.3%)	3214 (11.1%)	3096 (11.4%)	3044 (11.4%)	2918 (11.0%)	2641 (10.8%)	2417 (10.3%)	1958 (10.2%)	43,974 (11.0%)
Northeastern	8342 (24.9%)	7330 (22.9%)	7119 (23.8%)	6771 (23.8%)	7018 (21.6%)	6183 (17.1%)	6205 (21.0%)	6281 (21.7%)	5864 (21.7%)	5936 (22.3%)	6076 (22.9%)	5599 (23.0%)	5149 (22.0%)	4778 (25.0%)	88,651 (22.3%)
Northern	6279 (18.7%)	5906 (18.5%)	5325 (17.8%)	4881 (17.2%)	5288 (16.3%)	5516 (15.2%)	4778 (16.2%)	4616 (15.9%)	4277 (15.8%)	4023 (15.1%)	4055 (15.3%)	3666 (15.1%)	3375 (14.4%)	2905 (15.2%)	64,890 (16.3%)
Southern	5174 (15.4%)	4561 (14.3%)	4099 (13.7%)	3791 (13.3%)	3687 (11.4%)	3638 (10.0%)	3226 (10.9%)	3157 (10.9%)	2997 (11.1%)	2829 (10.6%)	2720 (10.3%)	2592 (10.6%)	2297 (9.8%)	1933 (10.1%)	46,701 (11.7%)
Western	1626 (4.8%)	1523 (4.8%)	1531 (5.1%)	1320 (4.6%)	1174 (3.6%)	1086 (3.0%)	1133 (3.8%)	1075 (3.7%)	1000 (3.7%)	960 (3.6%)	985 (3.7%)	903 (3.7%)	883 (3.8%)	682 (3.6%)	15,881 (4.0%)
Not recorded	158 (0.5%)	95 (0.3%)	94 (0.3%)	105 (0.4%)	89 (0.3%)	116 (0.3%)	80 (0.3%)	57 (0.2%)	65 (0.2%)	57 (0.2%)	53 (0.2%)	68 (0.3%)	1215 (5.2%)	658 (3.4%)	2910 (0.7%)
**Median (IQR) days to initiate ART**	81 (20–630)	63 (15–485)	50 (14–342)	41 (9–266)	41 (7–242)	54 (10–618)	36 (9–206)	31 (9–119)	30 (10–98)	28 (9–84)	23 (7–65)	21 (6–58)	21 (5–56)	14 (2–35)	35 (8–175)
Permanently lost	664 (2.0%)	666 (2.1%)	745 (2.5%)	781 (2.7%)	1024 (3.2%)	1126 (3.1%)	840 (2.8%)	695 (2.4%)	551 (2.0%)	406 (1.5%)	351 (1.3%)	211 (0.9%)	120 (0.5%)	29 (0.2%)	8209 (2.1%)
**Vital status**															
Alive	24,018 (71.6%)	23,037 (72.1%)	21,920 (73.3%)	21,354 (75.1%)	25,349 (78.2%)	29,431 (81.2%)	24,026 (81.4%)	24,280 (83.7%)	23,165 (85.6%)	23,300 (87.4%)	23,537 (88.8%)	21,885 (89.9%)	21,463 (91.8%)	17,649 (92.3%)	324,414 (81.5%)
AIDS-related COD	4927 (14.7%)	4783 (15.0%)	4397 (14.7%)	3937 (13.8%)	3921 (12.1%)	3733 (10.3%)	3164 (10.7%)	2765 (9.5%)	2309 (8.5%)	2018 (7.6%)	1782 (6.7%)	1501 (6.2%)	1165 (5.0%)	952 (5.0%)	41,354 (10.4%)
Non-AIDS related COD	4134 (12.3%)	3707 (11.6%)	3262 (10.9%)	2907 (10.2%)	2855 (8.8%)	2821 (7.8%)	2168 (7.3%)	1794 (6.2%)	1473 (5.4%)	1227 (4.6%)	1098 (4.1%)	878 (3.6%)	687 (2.9%)	462 (2.4%)	29,473 (7.4%)
Unknown COD	477 (1.4%)	437 (1.4%)	343 (1.1%)	250 (0.9%)	297 (0.9%)	262 (0.7%)	171 (0.6%)	164 (0.6%)	126 (0.5%)	118 (0.4%)	90 (0.3%)	90 (0.4%)	63 (0.3%)	53 (0.3%)	2941 (0.7%)
**Median (IQR) age at death [years]**															
AIDS-related	39.5 (33.6–46.7)	40.0 (33.4–46.4)	39.5 (33.3–46.5)	39.4 (33.1–46.8)	39.5 (33.2–46.7)	39.7 (33.1–47.0)	39.7 (31.9–47.6)	39.8 (31.6–47.8)	39.8 (31.8–48.0)	40.1 (31.6–48.0)	40.3 (32.1–48.1)	40.4 (32.0–49.1)	41.0 (31.6–49.6)	41.9 (33.2–50.1)	39.8 (32.9–47.2)
Non-AIDS-related	43.7 (37.4–51.8)	43.5 (36.8–51.3)	43.5 (36.9–51.7)	43.6 (36.4–51.7)	43.6 (36.3–51.7)	43.5 (36.3–51.6)	44.1 (36.3–51.9)	43.4 (35.1–51.4)	43.3 (35.1–52.0)	43.0 (34.1–52.1)	43.3 (34.3–52.1)	43.1 (33.3–51.4)	44.6 (36.5–52.3)	45.2 (33.4–53.5)	43.6 (36.2–51.8)
**Median (IQR) years to death**															
AIDS-related	2.6 (0.4–7.3)	2.5 (0.4–7.0)	2.3 (0.3–6.6)	2.3 (0.3–6.2)	2.1 (0.3–5.7)	1.6 (0.2–5.2)	1.3 (0.2–4.5)	1.4 (0.2–4.2)	1.1 (0.2–3.6)	0.9 (0.2–3.0)	0.6 (0.1–2.3)	0.5 (0.1–1.8)	0.4 (0.1–1.3)	0.2 (0.1–0.8)	1.3 (0.2–4.5)
Non-AIDS-related	4.4 (0.9–9.4)	4.7 (1.1–9.0)	4.2 (0.9–8.3)	3.9 (0.9–7.8)	3.9 (0.8–7.3)	3.3 (0.6–6.6)	3.0 (0.7–5.9)	2.7 (0.6–5.3)	2.3 (0.5–4.4)	1.8 (0.4–3.7)	1.4 (0.3–3.0)	1.0 (0.2–2.3)	0.7 (0.2–1.4)	0.3 (0.1–0.7)	2.7 (0.6–6.2)

### Cause of death classification accuracy

A group of clinicians and biostatisticians compared free-text COD and algorithm classified COD of 544 randomly selected cohort participants who died over the 15-year study period. Participants in each of the COD groups were represented. The accuracy rate of the algorithm classification was 98% (532/544 cases).

### Cause of death

**Fig. 2:**
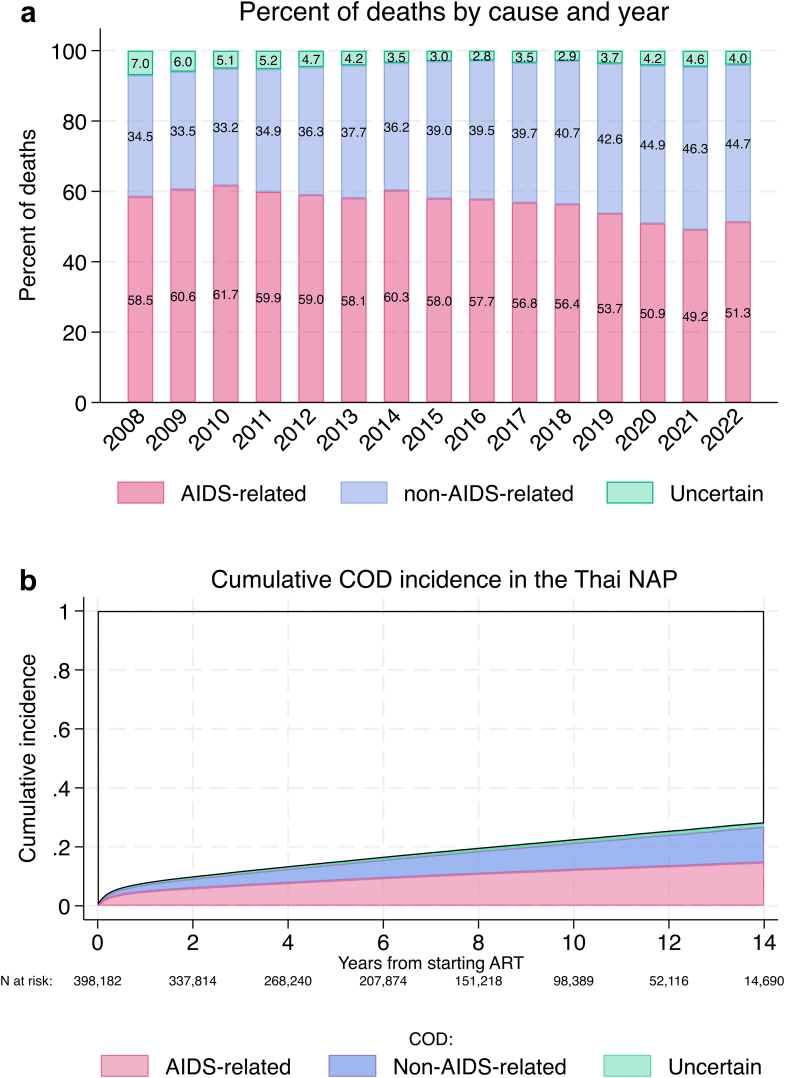
a) AIDS-related, non-AIDS related and uncertain deaths as a percentage of total deaths from 2008 to 2022. Since study follow-up was censored in January 2023, this year is omitted from the graph. b) Cumulative incidence of AIDS-related, non-AIDS-related and uncertain COD in people enrolling to the Thai NAP who started ART.

Median (IQR) duration of follow-up since ART initiation was 6.3 (3.0–10.0) years. Over 2,631,435 person years of follow-up (PY) 73,768 (18.5%) participants died. The highest proportion died of AIDS-related COD (41,354/73,768 (56%)), followed by non-AIDS-related COD (n = 29,473 (40%)) and 2941’s (4%) COD were uncertain. The median time from ART start to AIDS-related death was 1.3 years, and median time to non-AIDS-related death was 2.7 years. AIDS-related deaths were 58% and 61.7% in 2008 and 2010, respectively; thereafter the percentage of AIDS-related deaths decreased to approximately 50% from 2020 onwards (Fig. 2A).

Specific causes of death for AIDS-related, non-AIDS-related and uncertain COD are shown in Supplementary Table S1. Of the 41,354 AIDS-related COD, the majority were unspecified (n = 15,871 (38.4%), followed by TB/mycobacteria (n = 6327 (15.3%) and infections other than Cryptococcus, PCP, pneumonia or mycoses (n = 6327 (15.3%). Of the 29,473 non-AIDS related deaths, the highest proportion were non-AIDS related infections excluding COVID-19 (n = 5857 (19.9%), followed by non-AIDS-related cancers (n = 4739 (16.1%) and cardiovascular disease (n = 4591 (15.6%).

Thirty-one percent (22,976/73,768) of deaths occurred before 6 months. Of 26,347 participants who died from AIDS-related causes after 6 months, the median (IQR) most recent viral load result in the previous year was 45 (20–82,094) copies/mL in 10,763, and 15,584 had no viral load recorded in the 12 months prior to death. Of 22,420 participants who died from non-AIDS-related causes after 6 months, the median (IQR) most recent viral load result in the previous year was 40 (20–42,622) copies/mL and 10,233 had no viral load recorded in the 12 months prior to death.

### Cause-specific cumulative incidence

The cumulative incidence of each COD with other causes as competing risks are displayed in Fig. 2B, and described in Supplementary Table S2. One year after starting ART, the cumulative mortality incidence was 4.78% (95% CI 4.72–4.85) for AIDS-related COD, and 2.39% (95% CI 2.34–2.44) for non-AIDS-related COD. AIDS-related mortality was highest in the earlier years but slowed down thereafter. In contrast, non-AIDS related COD increased more rapidly with increasing follow-up (Fig. 2B, Supplementary Table S2). At 14 years, the cumulative incidence of uncertain COD was 1.16% (95% CI 1.11–1.21). The cumulative incidence of AIDS-related mortality was 14.74% (95% CI 14.56–14.91), non-AIDS mortality was 12.04% (95% CI 11.86–12.21), and of all-cause mortality was 27.93% (95% CI 27.69–28.17).

**Supplementary Fig. S1:**
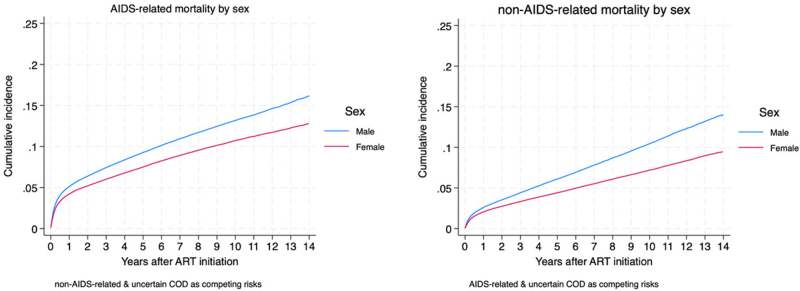


**Supplementary Fig. S2:**
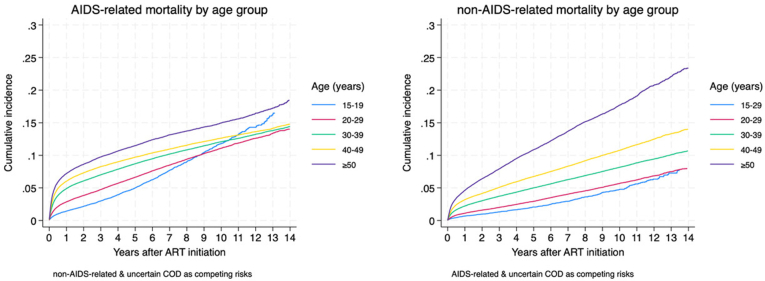


**Supplementary Fig. S3:**
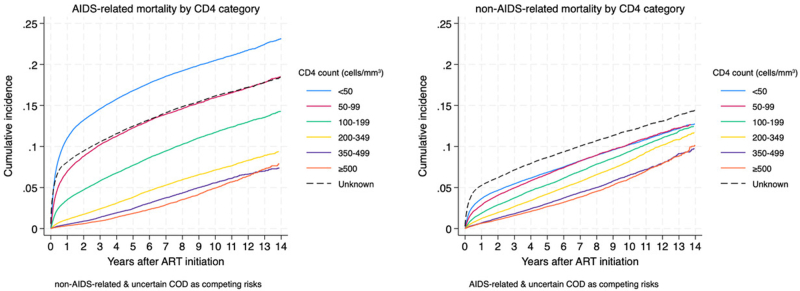


**Supplementary Fig. S4:**
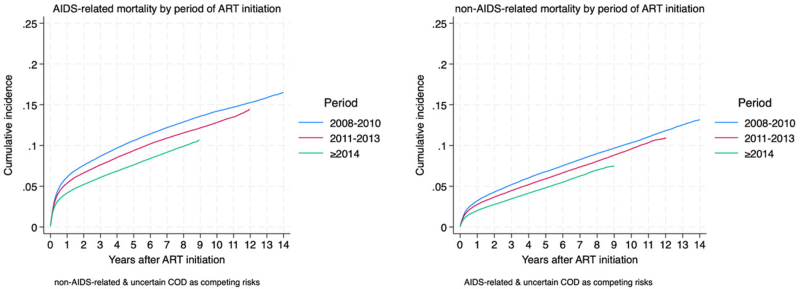


**Supplementary Fig. S5:**
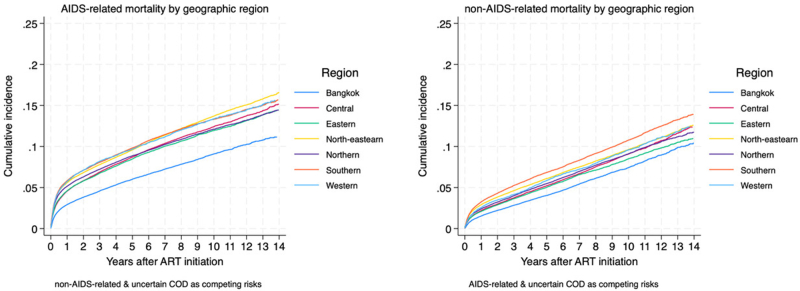


Both AIDS and non-AIDS cumulative mortality incidences were higher in males than females (Supplementary Fig. S1). Non-AIDS mortality cumulative incidence showed a strong relationship with increasing age: this relationship was apparent with AIDS-related mortality in the early follow-up period, but the mortality rate among those aged <30 years increased more rapidly and approached the incidence of those who initiated ART at the age of 30–49 years in later follow-up years, although the absolute number of PLHIV aged 15–19 years was low (Supplementary Fig. S2). Increasing CD4 counts at baseline were associated with lower AIDS-related mortality over the entire follow-up period. There was also a relationship with non-AIDS-related mortality, although differentiation across adjacent CD4 strata was not as pronounced. In those without CD4 counts in the window defined by baseline, the CI of AIDS-related mortality was approximately equal to those with baseline CD4 cell counts of 50–99 cells/mm^3^, but had a cumulative incidence of non-AIDS-related mortality higher than any other CD4 cell count strata (Supplementary Fig. S3). There was a period effect evident, with later cohort entry associated with a lower CI for both AIDS- and non-AIDS-related cumulative incidence (Supplementary Fig. S4). Compared to those who received care in the greater Bangkok metropolis, those who received care in other geographic regions had a higher cumulative incidence of AIDS- and non-AIDS mortality (Supplementary Fig. S5).

### Adjusted competing risk models

**Fig. 3:**
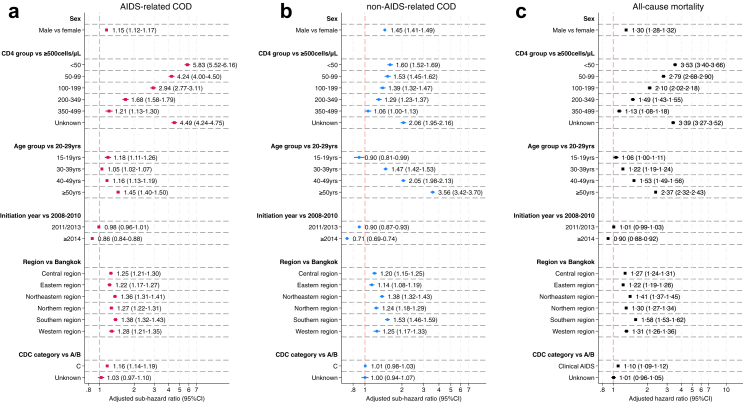
Adjusted sub-hazard ratios (95% CI) for a) AIDS-related COD, b) non-AIDS-related COD, and c) adjusted hazard ratios (95% CI) for all-cause mortality for people enrolling to the Thai NAP from 2008 to 2021 who started ART. Non-AIDS-related and uncertain COD are competing risks for AIDS-related COD, and AIDS-related and uncertain COD are competing risks for non-AIDS-related COD.

**Supplementary Fig. S6:**
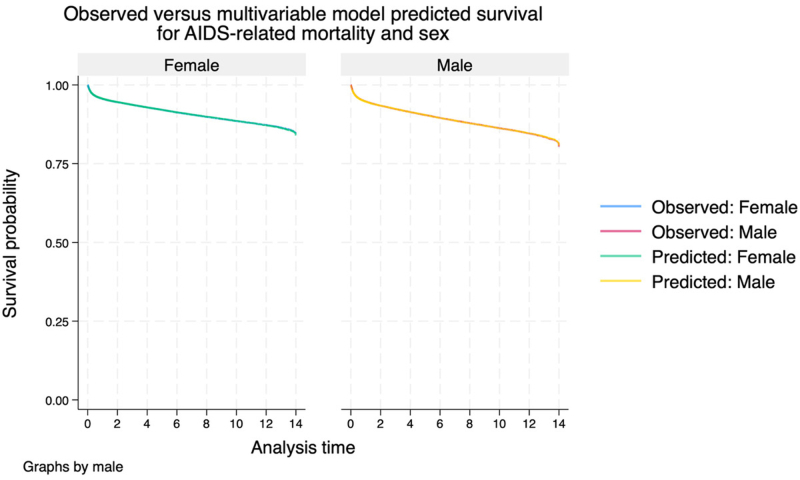
Figure S6. Observed versus multivariable model predicted survival for AIDS-related mortality and sex.

**Supplementary Fig. S7:**
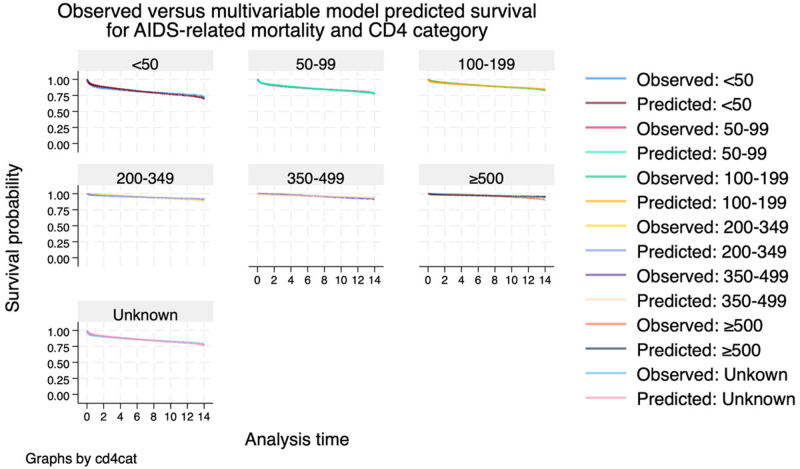
Figure S7. Observed versus multivariable model predicted survival for AIDS-related mortality and CD4 category

**Supplementary Fig. S8:**
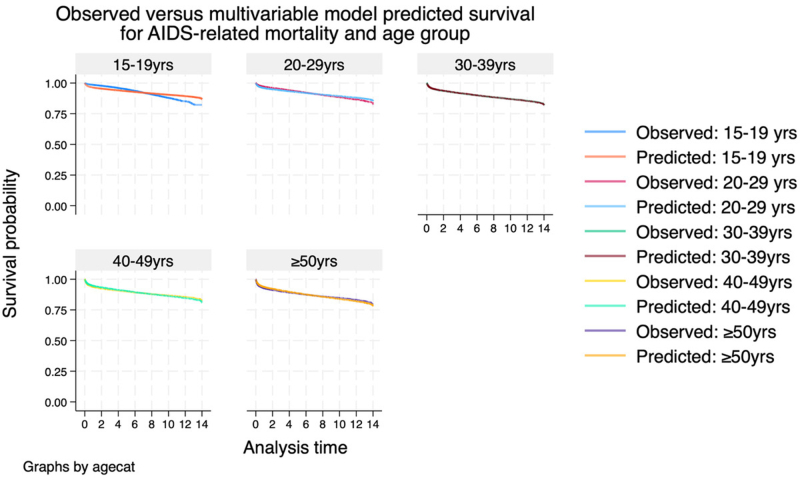
Figure S8. Observed versus multivariable model predicted survival for AIDS-related mortality and age group.

**Supplementary Fig. S9:**
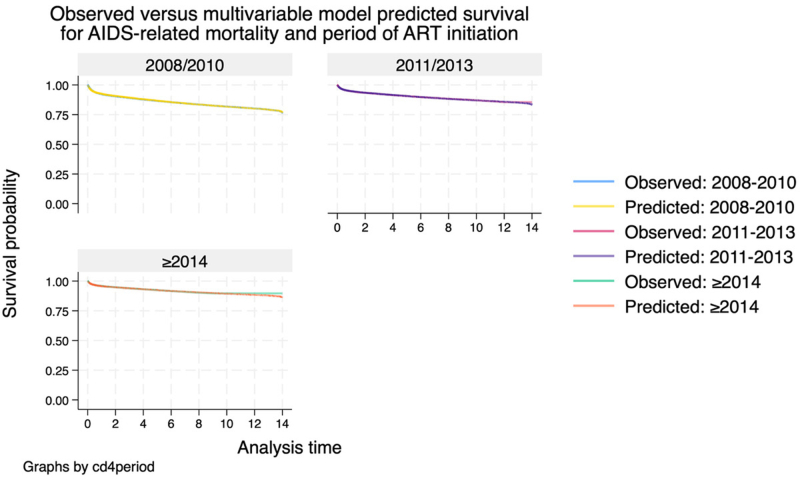
Figure S9. Observed versus multivariable model predicted survival for AIDS-related mortality and period of ART initiation.

**Supplementary Fig. S10:**
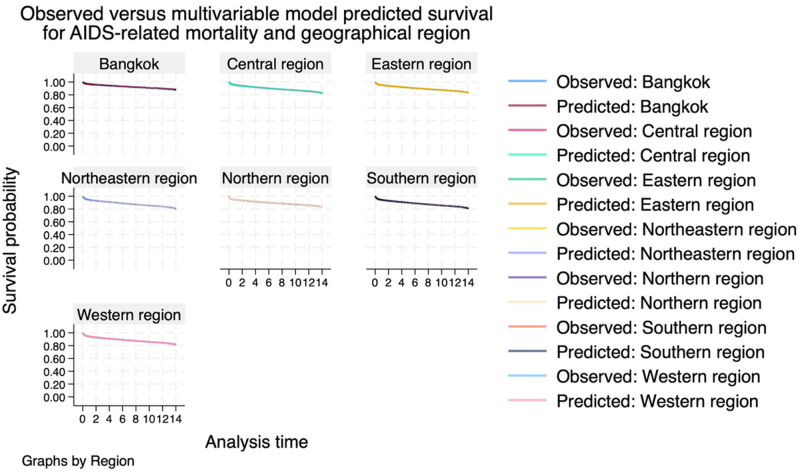
Figure S10. Observed versus multivariable model predicted survival for AIDS-related mortality and geographical region.

**Supplementary Fig. S11:**
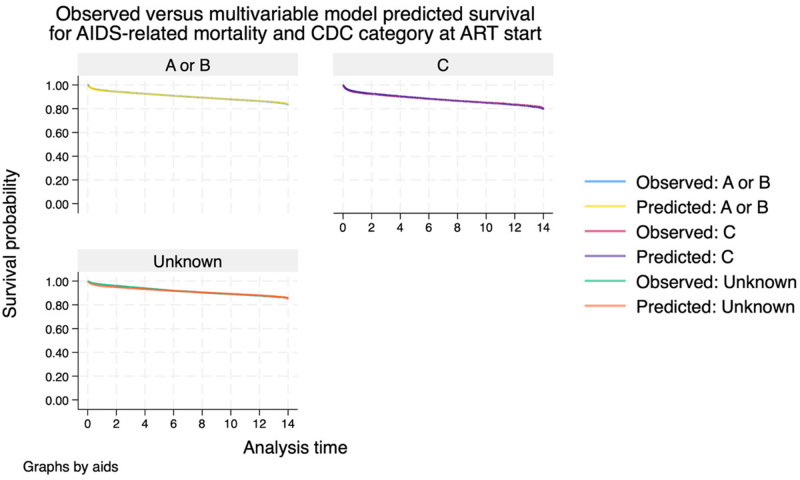
Figure S11. Observed versus multivariable model predicted survival for AIDS-related mortality and CDC category at ART start.

**Supplementary Fig. S12:**
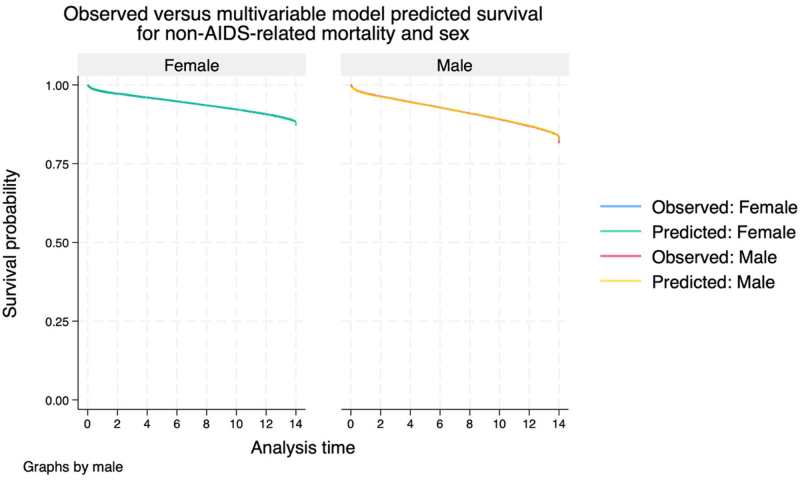
Figure S12. Observed versus multivariable model predicted survival for non-AIDS-related mortality and sex.

**Supplementary Fig. S13:**
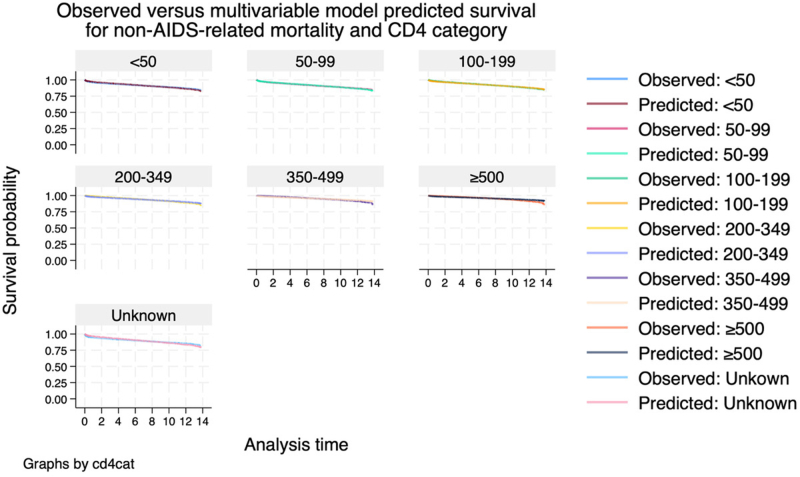
Figure S13. Observed versus multivariable model predicted survival for non-AIDS-related mortality and CD4 category.

**Supplementary Fig. S14:**
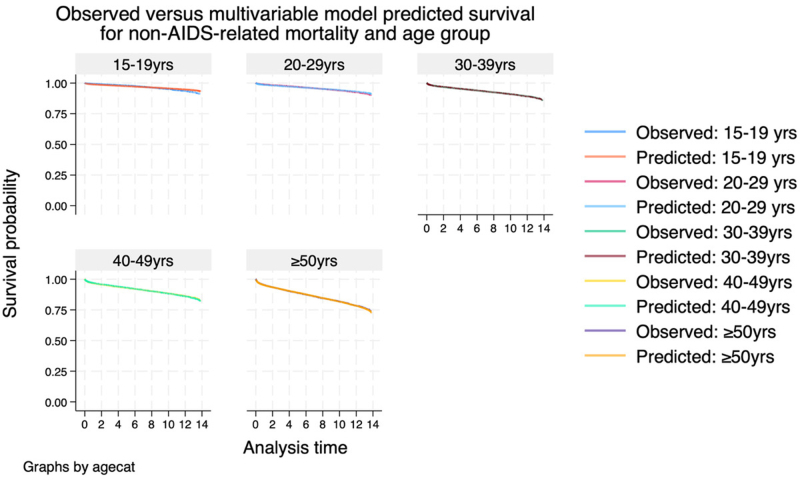
Figure S14. Observed versus multivariable model predicted survival for non-AIDS-related mortality and age group.

**Supplementary Fig. S15:**
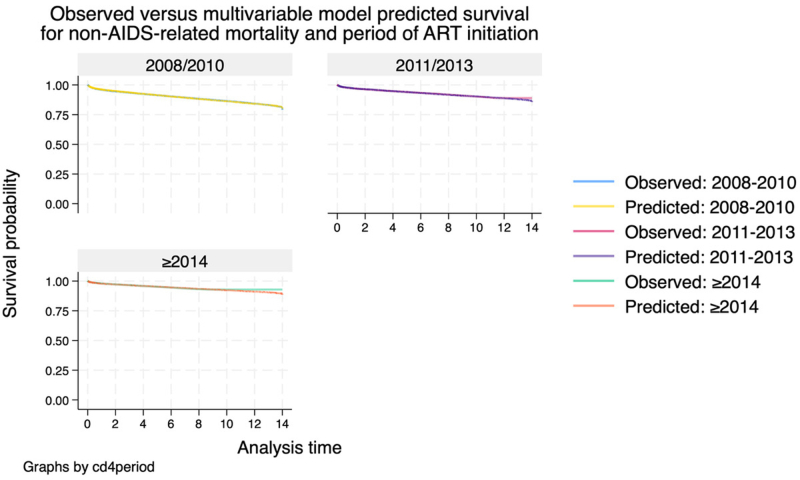
Figure S15. Observed versus multivariable model predicted survival for non-AIDS-related mortality and period of ART initiation.

**Supplementary Fig. S16:**
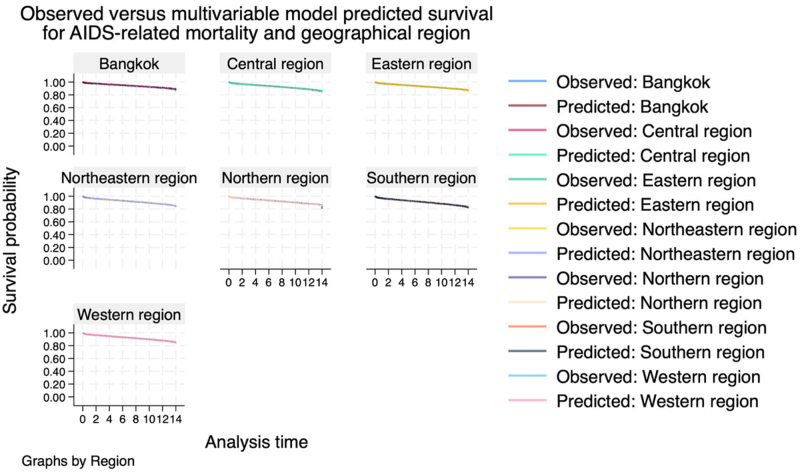
Figure S16. Observed versus multivariable model predicted survival for non-AIDS-related mortality and geographical region.

**Supplementary Fig. S17:**
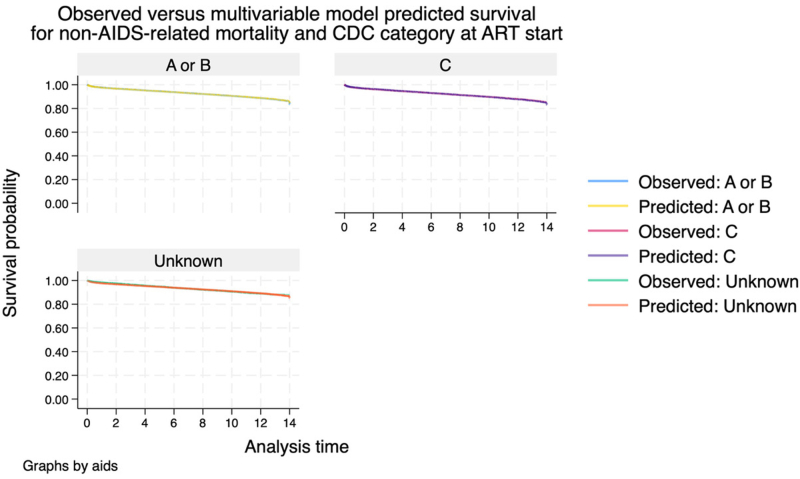
Figure S17. Observed versus multivariable model predicted survival for non-AIDS-related mortality and CDC category at ART start.

The patterns observed in the univariable cumulative incidence plots were largely consistent with the adjusted sHR for AIDS- and non-AIDS-related COD from competing risks regression models (Fig. 3). Males had significantly higher risk for both AIDS- and non-AIDS-related mortality compared to the females. Youths aged 15–19 years had a higher risk of AIDS-related mortality, but lower risk of non-AIDS-related mortality compared to those aged 20–29 years; and the risk for both AIDS- and non-AIDS-related mortality increased in relative to the 20–29 age group in those aged ≥30. Low baseline CD4 count showed the highest risk of AIDS-related mortality which attenuated with increasing CD4 cell counts (sHR of 5.83, 4.24 and 2.94 for those with CD4 cell counts <50, 50–99 and 100–199 cells/mm^3^ respectively, compared to those with CD4 counts 500 cells/mm^3^ as a reference). The sub-hazard ratio for AIDS-related mortality was significantly higher in each CD4 count stratum compared to those with baseline CD4 counts ≥500 cells/mm^3^. The same pattern was noted with non-AIDS-related mortality, but the magnitude of the increases compared to those with CD4 counts ≥500 cells/mm^3^ was reduced compared to the risk of AIDS-related mortality, and the risk for non-AIDS-related mortality in those with CD4 counts from 350 to 499 cells/mm^3^ remained elevated but not significantly higher. Mortality rates decreased with more recent cohort entry and as expected, being permanently lost from care significantly increased the adjusted risk of AIDS- and non-AIDS mortality. The adjusted risk of AIDS-related mortality ranged from an increase 22–38% in areas outside the capital city; the non-AIDS-related risk ranged from 14 to 53% higher than observed in Bangkok. Our plots of observed versus multivariable predicted survival curves found adequate concordance, and the observed versus predicted survival curves for AIDS- and non-AIDS-related mortality are presented in Supplementary Figs. S6–S17.

### Standardized mortality ratios for PLHIV against the general Thai population

The SMR for the entire cohort was 6.62 (95% CI 6.57–6.66). Females had a higher SMR compared to the males (9.08 versus 5.83), and SMR decreased with increasing age and increasing CD4 cell counts. We further explored sex differences in SMR by age category. In females aged 15–19 years and 20–29 years, the SMR was approximately 3-fold higher than in males in the same age category. The magnitude of the relative difference in SMR in females versus males of equivalent ages decreased with increasing age (Table 2).

**Table 2:** Standardized mortality ratios (SMR) for PLHIV versus the general Thai population, overall and by sex and age group.

Group	SMR (95% CI)		
	Overall	Females	Males
Overall	6.62 (6.57–6.66)	9.08 (8.97–9.20)	5.83 (5.78–5.88)
Age group			
15–19 years	15.5 (14.25–16.86)	30.37 (26.89–34.30)	10.76 (9.59–12.08)
20–29 years	13.86 (13.58–14.14)	30.93 (29.87–32.04)	10.87 (10.61–11.14)
30–39 years	11.03 (10.87–11.17)	18.66 (18.249–19.08)	9.12 (8.98–9.27)
40–49 years	6.36 (6.28–6.44)	8.84 (8.65–9.05)	5.62 (5.53–5.71)
50–59 years	4.20 (4.12–4.27)	5.17 (5.01–5.34)	3.84 (3.76–3.93)
≥60 years	3.00 (2.92–3.08)	3.41 (3.26–3.56)	2.82 (2.72–2.92)

## Discussion

In this analysis of 398,192 PLHIV enrolled into the Thai National AIDS Program (NAP) from 2008 to 2021 who started ART, the cumulative mortality incidence at 14 years was 27.93% (27.69–28.17). Most deaths were AIDS-related (14.74%), followed by non-AIDS-related (12.04%) and 1.16% of the deaths could not be definitively classified as AIDS- or non-AIDS-related.

Lower CD4 counts were associated with the highest relative risk of AIDS-related mortality, which is consistent with reports from other large cohorts.^4^^,^^7^^,^^9^ WHO defines late HIV presentation in adults living with HIV as presenting with CD4 cell counts <200 cells/mm^3^ or WHO stage 3 and 4, while a recent consensus from the EuroTEST HIV Late Diagnosis Definition Working Group expanded this definition to include CD4 counts <350 cells/mm^3^ or an AIDS-defining event, regardless of CD4 count, after excluding people diagnosed during acute HIV infection.^14^ Although we were unable to definitively exclude individuals presenting to care in the acute phase of infection, applying recent consensus CD4 count criteria alone classified 61.0% of the cohort as late-presenters. Those presenting with CD4 counts <350 cells/mm^3^ had a significantly increased risk of AIDS-related mortality. The risk of non-AIDS-related mortality was also elevated in these late presenters. These findings are consistent with the START study that demonstrated uncontrolled viral replication and immune activation, even at high CD4 counts, is associated with both short-term and persistent deleterious effects on health.^15^^,^^16^

An additional 14.6% of our study cohort did not have an available CD4 count within the window used to define baseline. Missing CD4 cell counts at baseline may be a surrogate for failure to completely engage with the health system, since these individuals had the highest cumulative incidence of non-AIDS-related mortality, and an adjusted relative risk of AIDS-related-mortality comparable to those with baseline CD4 counts from 50 to 99 cells/mm^3^. Hospitals in rural areas do not always have the capacity to measure CD4 cell counts and send blood samples for testing to urban centres. In such settings, CD4 count results may not be known for a month, so late presenters can die before CD4 results are known.

Western cohorts have demonstrated a shift from AIDS-related to non-AIDS-related mortality in the modern ART era,^4^^,^^5^ but the shift in our cohort was less dramatic with a reduction in AIDS-related-mortality from approximately 60% of deaths from 2008 to 2016, then reducing to approximately 50% of deaths from 2020 onwards. The cumulative incidence of AIDS-related deaths in our study after 3 months was 2.74%, and this increased by around 1% at 6 months, and again at 12, 24 and 36 months. Thereafter AIDS-related deaths slowly declined until year 8, then fluctuated from approximately 0.6 to 0.7% per year. High rates of AIDS-related deaths early after ART initiation was related to treatment initiation at low CD4 counts. Continued deaths from AIDS-related causes, even after several years of ART, most likely represents poor adherence to treatment or disengagement from care, as losses to follow-up are a strong predictor of subsequent mortality in people taking ART in the Thai NAP.^17^^,^^18^ Access to second-line regimens was available at all hospitals, in every province of Thailand for the duration of the current study. Non-AIDS-related COD incidence increased more slowly than AIDS-related COD in the first 2 years, then began to increase at rates of approximately 0.7–0.8% per year thereafter. Possible reasons for the less dramatic change to non-AIDS related mortality in our cohort were the comparatively low CD4 cell counts at ART initiation, and the comparatively younger age distribution of individuals in our cohort versus Western cohorts.^7^

Amongst the AIDS-related deaths that could be classified, the majority were from tuberculosis. This is consistent with studies in other high burden TB settings,^19^^,^^20^ and highlights the importance of prompt investigation of possible active TB disease in PLHIV, and initiation of tuberculosis preventive therapy for those without active TB disease. WHO advocates rapid (within 7 days) ART initiation following confirmed diagnosis and clinical assessment, and strongly recommends same day ART initiation unless there are clinical reasons to delay treatment.^21^ The benefits of rapid and same day ART include a reduction in deaths from opportunistic infections.^22^^,^^23^ The substantial numbers of PLHIV who initiated treatment at low CD4 counts highlights the importance of adequately assessing patients for their risk of opportunistic infections and following them up for evidence of immune recovery syndrome, particularly those who initiate ART with CD4 cell counts ≤100 cells/mm^3^.

Despite universal access for PLHIV in Thailand, those outside the greater Bangkok metropolis had significantly higher rates of AIDS- and non-AIDS related deaths to those living in the capital. This is partly related to CD4 counts at ART start: median CD4 counts were 300 cells/mm^3^ in Bangkok, but <200 cells/mm^3^ in all other regions. It also likely relates to inequalities between people living in the capital city and those living in other regions, reflecting economic and educational opportunity disparities across Thailand. In support of this, Bangkok which has the highest GDP per capita, and in 2020 the average income in Bangkok was approximately double that of the central region, and more than 6.5 times higher than the northeast region.^24^

Adolescents aged 15–19 years were at the high risk of early AIDS-related mortality which continued to increase during the study. Adolescents and young adults find maintaining adherence to ART challenging, and this problem can be exacerbated by stigma and mental health issues. We have previously reported that in the NAP, retention among youth aged 15–19 with behaviourally acquired HIV is worse than among youth aged 20–24 or those with vertically acquired HIV.^25^

In our adjusted analyses, the risk of mortality in men versus women for AIDS- and non-AIDS-related causes was 21% and 49% higher, respectively. However, despite this, the age standardized relative mortality for PLHIV compared to the general population was much higher in women (9.08) compared to men (5.83). Moreover, the SMR was approximately 3 times higher compared to men, in young women aged 15–19 and 20–29 years, and remained elevated across all age groups. This indicates that while men have a higher absolute risk of mortality from specific causes, the overall impact of HIV on mortality relative to the general population is more severe for women in general, and younger women in particular. Sex-disparities in HIV health outcomes and healthcare utilization have been described in a number of disease states including HIV.^26^, ^27^, ^28^ Higher SMR in female (8.8) versus male PLHIV (4.9) was also observed in a National UK cohort study between 2008 and 2012.^29^ A number of factors may contribute to the alarmingly higher SMR in women, particularly younger women, in this cohort. Biological differences in inflammatory pathways may play a role,^30^ but there are challenges relating to tolerance to some ART classes, pregnancy and motherhood, stigma and discrimination, mental health and support services, and policy and programmatic gaps which fail to target women living with HIV.^17^^,^^31^ These factors need urgent exploration to address factors which contribute to poor health outcomes in women.

In response to the high numbers of AIDS related deaths and high numbers of people presenting with advanced HIV, HIV-self testing was introduced into the universal health coverage program in 2023, and same day or rapid ART initiation is now standard of care and has been shown to improve outcomes in adolescents in the NAP.^32^ In addition, to improve detection of TB, the country has expanded the use of molecular diagnosis including GeneXpert MTB/RIF, urine lipoarabinomannan (urine LF-LAM) and chest x-rays for all PLHIV were introduced into the NAP program in 2021. Additionally, cryptococcal Ag (CrAg) screening was introduced for individuals with CD4 cell count <100 cells/mm^3^ to facilitate early detection and management of cryptococcal disease. Furthermore, tuberculosis preventive therapy was integrated in the National guideline since 2024, with a particular emphasis on individuals newly diagnosis with HIV and those who have been on ART for less than 12 months, regardless of CD4 cell counts.

There are several limitations in our study. First, although our all-cause mortality estimates are robust because deaths of all PLHIV enrolled into the NAP are obtained real time through death-registry linkage, there is a possibility that the COD recorded on the death certificate led to misclassification of AIDS-related-deaths as non-AIDS-related, and vice versa. Second, the NAP database is an administrative system, so some data elements are missing or incomplete, but to minimize participant losses from the data analyses, we included ‘unknown’ categories in our multivariable regression models. Third, we were unable to model risk group for HIV acquisition, as policies targeting key populations were introduced in 2015, and self-reported risk is only recorded in the Voluntary Counselling and Testing (VCT) database, not the treatment database. Fourth, as an observational study, the results are subject to unobserved confounding factors. However, despite these limitations, this report is the largest study documenting COD after ART initiation, in a national cohort of patients treated according to the national treatment guidelines which also assesses the death in the death registry. It underscores the importance of timely ART initiation and comprehensive care strategies in initiating ART and managing HIV, and demonstrates further efforts are needed to ensure early diagnosis and equitable health outcomes for women, and people living in all regions of the country.

In conclusion, this study highlights the shifting mortality patterns among PLHIV who initiated ART through the National AIDS Program in Thailand. While ART has significantly reduced AIDS-related-mortality over time, non-AIDS-related deaths, particularly from non-communicable diseases (NCDs), are increasingly prevalent. Moving forward, healthcare strategies must focus on early diagnosis, timely ART initiation, and holistic care that integrates both infectious and NCD management. Additionally, addressing disparities in outcomes, particularly for women and individuals living in rural or underserved areas, will be crucial in achieving equitable health outcomes for all PLHIV.

## Contributors

CL, SK, SN, NP, PC, KR and AA conceptualised the study. CL, SN, PN, NP, NH, PC, OP, KR and AA guided the study. SK performed the statistical analysis with support from TA and JS. CL and SK wrote the first draft of the manuscript, and all authors critically reviewed the manuscript and approved the final version for publication. CL and SK have access to all the study data and take responsibility for integrity of the data reported and the accuracy of the data analysis.

## Data sharing statement

The datasets generated and analysed during the current study are not publicly available due to the need to protect privacy and confidentiality. Data is available on request conditional on approval of the Thai Ministry of Public Health and National Health Security Organization.

## Declaration of interests

AA has received grants from Gilead Science, ViiV/GSK, Roche, MSD, and Janssen Research & Development which went directly to the institution; received transportation support from Gilead Sciences to present poster and attend CROI 2025; and non-financial interests to work for 1) Strategic and Technical Advisory group to WHO for HIV/hepatitis/STI, 2) Thai AIDS society committee, and 3) Thailand National ART, TB, HIV, hepatitis program committee. The rest of the authors declare no conflict of interest.
