# Supplementary material for: Cause of death in people living with HIV who initiated antiretroviral therapy after enrolling to the Thai National AIDS Program from 2008 to 2021
Source: Lancet Reg Health Southeast Asia. 2025 Apr 23;36:100576. doi: 10.1016/j.lansea.2025.100576 (PMC12051708; doi:10.1016/j.lansea.2025.100576)
Supplement: Supplementary Table S1 [file mmc1.docx]

**Table S1. Cause of death in PLHIV starting treatment after enrolment to the Thai National AIDS Program from 2008-2021.**

| **Broad category** | **Specific category** | **Female** | **Male** | **Total** |
| --- | --- | --- | --- | --- |
| AIDS-related  (N=41,354) | Unspecified AIDS-related | 5,339 (37·7%) | 10,532 (38·7%) | 15,871 (38·4%) |
|  | Tuberculosis/Mycobacteria | 1,792 (12·7%) | 4,535 (16·7%) | 6,327 (15·3%) |
|  | Other infections | 2,210 (15·6%) | 4,117 (15·1%) | 6,327 (15·3%) |
|  | PCP | 1,554 (11·0%) | 2,861 (10·5%) | 4,415 (10·7%) |
|  | Pneumonia | 1,227 (8·7%) | 2,363 (8·7%) | 3,590 (8·7%) |
|  | Mycoses | 781 (5·5%) | 1,420 (5·2%) | 2,201 (5·3%) |
|  | AIDS-defining cancers | 882 (6·2%) | 643 (2·4%) | 1,525 (3·7%) |
|  | Cryptococosis | 343 (2·4%) | 703 (2·6%) | 1,046 (2·5%) |
|  | Wasting Syndrome | 20 (0·1%) | 32 (0·1%) | 52 (0·1%) |
| Non-AIDS-related  (N=29,473) | non-AIDS infection | 2,186 (23·3%) | 3,671 (18·3%) | 5,857 (19·9%) |
|  | non-AIDS cancer | 1,593 (17·0%) | 3,146 (15·6%) | 4,739 (16·1%) |
|  | Cardiovascular Disease | 1,433 (15·3%) | 3,158 (15·7%) | 4,591 (15·6%) |
|  | Pulmonary Diseases | 847 (9·0%) | 1,772 (8·8%) | 2,619 (8·9%) |
|  | Other | 686 (7·3%) | 1,686 (8·4%) | 2,372 (8·0%) |
|  | Liver disease | 551 (5·9%) | 1,579 (7·9%) | 2,130 (7·2%) |
|  | Accident/Injury | 371 (4·0%) | 1,423 (7·1%) | 1,794 (6·1%) |
|  | Urogenital Disease | 600 (6·4%) | 1,036 (5·2%) | 1,636 (5·6%) |
|  | Neurological Disease | 445 (4·8%) | 1,033 (5·1%) | 1,478 (5·0%) |
|  | Psychological Disease/Suicide | 157 (1·7%) | 638 (3·2%) | 795 (2·7%) |
|  | Diabetes Mellitus | 214 (2·3%) | 341 (1·7%) | 555 (1·9%) |
|  | Covid-19 | 123 (1·3%) | 304 (1·5%) | 427 (1·4%) |
|  | Hematologic Disease | 98 (1·0%) | 92 (0·5%) | 190 (0·6%) |
|  | Substance use | 17 (0·2%) | 145 (0·7%) | 162 (0·5%) |
|  | Assault | 12 (0·1%) | 64 (0·3%) | 76 (0·3%) |
|  | Endocrine Disease | 33 (0·4%) | 19 (0·1%) | 52 (0·2%) |
| Uncertain  (N=2,941) | Undocumented AIDS-related | 690 (74·2%) | 1,571 (78·1%) | 2,261 (76·9%) |
|  | Ill-defined old age | 177 (19·0%) | 347 (17·3%) | 524 (17·8%) |
|  | Probable AIDS-related | 63 (6·8%) | 93 (4·6%) | 156 (5·3%) |
