# Supplementary material for: Cause of death in people living with HIV who initiated antiretroviral therapy after enrolling to the Thai National AIDS Program from 2008 to 2021
Source: Lancet Reg Health Southeast Asia. 2025 Apr 23;36:100576. doi: 10.1016/j.lansea.2025.100576 (PMC12051708; doi:10.1016/j.lansea.2025.100576)
Supplement: Supplementary Table S2 [file mmc2.docx]

**Table S2. Cumulative mortality incidence (95%CI) after initiating ART expressed as a percentage, by cause of death.**

| **Years after ART** | **COD cumulative incidence (95%CI)** | | | |
| --- | --- | --- | --- | --- |
|  | **AIDS-Related** | **Non-AIDS-related** | **Uncertain** | **All-cause** |
| 0·25 | 2·74 (2·69 - 2·79) | 1·25 (1·21 - 1·28) | 0·16 (0·15 - 0·17) | 4·15 (4·09 - 4·21) |
| 0·5 | 3·77 (3·71 - 3·83) | 1·77 (1·73 - 1·81) | 0·23 (0·22 - 0·25) | 5·77 (5·7 - 5·85) |
| 0·75 | 4·35 (4·29 - 4·42) | 2·11 (2·06 - 2·15) | 0·27 (0·26 - 0·29) | 6·73 (6·66 - 6·81) |
| 1 | 4·78 (4·72 - 4·85) | 2·39 (2·34 - 2·44) | 0·3 (0·28 - 0·32) | 7·47 (7·39 - 7·56) |
| 1·5 | 5·45 (5·38 - 5·52) | 2·85 (2·8 - 2·9) | 0·35 (0·33 - 0·37) | 8·64 (8·56 - 8·73) |
| 2 | 5·95 (5·88 - 6·02) | 3·24 (3·18 - 3·29) | 0·39 (0·37 - 0·41) | 9·57 (9·48 - 9·67) |
| 3 | 6·9 (6·82 - 6·98) | 4 (3·94 - 4·06) | 0·45 (0·43 - 0·47) | 11·35 (11·25 - 11·45) |
| 4 | 7·77 (7·68 - 7·85) | 4·73 (4·67 - 4·8) | 0·51 (0·49 - 0·54) | 13·02 (12·91 - 13·12) |
| 5 | 8·6 (8·51 - 8·69) | 5·45 (5·37 - 5·52) | 0·58 (0·55 - 0·6) | 14·62 (14·51 - 14·74) |
| 6 | 9·41 (9·31 - 9·51) | 6·17 (6·09 - 6·25) | 0·64 (0·61 - 0·66) | 16·22 (16·09 - 16·34) |
| 7 | 10·16 (10·06 - 10·26) | 6·91 (6·83 - 7) | 0·7 (0·67 - 0·73) | 17·77 (17·64 - 17·91) |
| 8 | 10·86 (10·75 - 10·97) | 7·65 (7·55 - 7·74) | 0·75 (0·72 - 0·78) | 19·27 (19·13 - 19·4) |
| 9 | 11·54 (11·42 - 11·65) | 8·37 (8·27 - 8·47) | 0·81 (0·78 - 0·84) | 20·72 (20·57 - 20·86) |
| 10 | 12·19 (12·07 - 12·31) | 9·1 (8·99 - 9·21) | 0·87 (0·84 - 0·91) | 22·16 (22·01 - 22·32) |
| 11 | 12·78 (12·65 - 12·9) | 9·87 (9·75 - 9·99) | 0·94 (0·9 - 0·98) | 23·59 (23·42 - 23·76) |
| 12 | 13·42 (13·29 - 13·56) | 10·62 (10·49 - 10·76) | 1·01 (0·96 - 1·05) | 25·05 (24·87 - 25·24) |
| 13 | 14·06 (13·91 - 14·21) | 11·38 (11·23 - 11·53) | 1·09 (1·04 - 1·14) | 26·53 (26·32 - 26·73) |
| 14 | 14·74 (14·56 - 14·91) | 12·04 (11·86 - 12·21) | 1·16 (1·11 - 1·21) | 27·93 (27·69 - 28·17) |
